# Postoperative Recovery of Visual Function after Macula-Off Rhegmatogenous Retinal Detachment

**DOI:** 10.1371/journal.pone.0099787

**Published:** 2014-06-13

**Authors:** Mathijs A. J. van de Put, Danna Croonen, Ilja M. Nolte, Wouter J. Japing, Johanna M. M. Hooymans, Leonoor I. Los

**Affiliations:** 1 Department of Ophthalmology, University of Groningen, University Medical Center Groningen, Groningen, the Netherlands; 2 Department of Epidemiology, University of Groningen, University Medical Center Groningen, Groningen, the Netherlands; 3 W.J. Kolff Institute, Graduate School of Medical Sciences, University of Groningen, Groningen, the Netherlands; Massachusetts Eye & Ear Infirmary, Harvard Medical School, United States of America

## Abstract

**Purpose:**

To determine which factors affect the recovery of visual function in macula off rhegmatogenous retinal detachment (RRD).

**Methods:**

In a prospective study of forty-five patients with a primary macula-off RRD of 24 hours to 6 weeks duration, the height of the macular detachment was determined by ultrasonography. At 12 months postoperatively, best corrected visual acuity (BCVA), contrast acuity, and color confusion indexes (CCI) were obtained.

**Results:**

Macular detachment was present for 2–32 (median 7) days before repair. A shorter duration of macular detachment was correlated with a better CCI saturé (p = 0.0026) and lower LogMAR BCVA (better Snellen visual acuity)(p = 0.012). Also, a smaller height of macular detachment was correlated with a lower LogMAR BCVA (p = 0.0034). A younger age and lower pre-operative LogMAR BCVA at presentation were both correlated with better postoperative contrast acuity in the total group (age: p = 1.7×10^−4^ and pre-operative LogMAR BCVA: p = 0.0034).

**Conclusion:**

Functional recovery after macula-off RRD is affected by the duration and the height of the macular detachment. Recovery of contrast acuity is also affected by age and BCVA at presentation.

**Meeting presentation:**

ARVO annual meeting 2013, May 7, Seattle, Washington, United States of America.

**Trial registration:**

trialregister.nl NTR839

## Introduction

Rhegmatogenous retinal detachment (RRD) with an incidence of 18.9/100,000 people per year in the Netherlands [1] is a potentially blinding ophthalmic pathology. [2]–[3] The treatment modality of RRD is surgical reattachment of the retina. [4] Contrary to the high anatomical success rate [2]–[3], [5]–[8] the prognosis for the recovery of visual acuity (VA) may be disappointing. Permanent functional damage is particularly observed if the macula is detached, [9]–[17] which occurs in about 50% of cases. [1]

Factors that may influence functional recovery after macula-off RRD include preoperative VA, [2] duration of macular detachment, [2], [9], [11], [18]–[22] height of macular detachment, [2], [15], [22]–[25] age, [2], [22] and refractive error. [22] Most studies consider only the recovery of visual acuity and lack data on other aspects of macular function such as contrast acuity and color vision. [26], [27], [29]

The timing of optimal surgical intervention remains inconclusive. Burton observed that postoperative VA was dependent on the preoperative duration of macular detachment in RRD patients. [9] However, he and others suggested that a delay in surgical repair between 24 hours and 7 to 9 days does not preclude good functional recovery after macula-off RRD [9], [11], [14] and should not be expected to have a significant effect on final visual outcome. [11], [14]

The absence of a relation between the duration of the macular detachment within the first week and functional recovery may be a misguided assumption, since the effect of the height of the macular detachment has not been considered in most studies. [2], [15], [22]–[25], [29] Also, such an assumption is not in line with animal experiments, in which a progressive loss of photoreceptor cells is seen already within the first week of retinal detachment, [15] and it has been demonstrated that photoreceptor cell degeneration increases as the height of the macular detachment is increased. [15], [16]

The few studies on the influence of the height and the duration of the macular detachment on the postoperative recovery of VA [23], [24] found that - within the first week of macular detachment - a lower height of macular detachment was associated with better postoperative visual acuity. Furthermore, previous studies relate to the outcomes for patients who have undergone “conventional” (buckling) surgery. [2], [9], [11], [14], [18]–[22] However, in recent years trans pars plana vitrectomy (TPPV) is often used as a primary procedure, which might affect postoperative recovery. [30] Finally, follow-up periods in these studies ranged from 3 to 24 months, [2], [9], [11], [14], [18]–[21], [23], [24] whereas it is known that postoperative VA can improve gradually, reaching a maximum value between 6–12 months postoperatively. [10], [31]

In order to gain a better insight in factors influencing the long term postoperative recovery of visual function after macula-off RRD, our study aimed to establish the relative contributions of a number of factors including both the duration and the height of the macular detachment to various aspects of visual recovery (i.e. best corrected visual acuity (BCVA), contrast acuity, and color vision).

## Materials and Methods

In this prospective observational study we studied patients with a first presentation of macula-off RRD who underwent successful reattachment surgery, after 24 hours to 6 weeks of macular detachment. The research protocol was approved by the University Medical Center Groningen (UMCG) review board ethics committee, and was carried out in accordance with the tenets of the declaration of Helsinki. The study was registered with the Dutch Trial Register (NTR839). All patients were operated on at the ophthalmology department of the UMCG. The study was carried out over a four year period (February 1, 2007 - February 1, 2011).

Adult patients visiting the ophthalmology department of the UMCG with a first presentation of RRD with a macular detachment of 24 hours to 6 weeks duration were invited to participate in this study. We defined the moment of macular detachment as the subjective loss of VA, since an objective method to determine this moment does not exist. Patients with a macular detachment of <24 hours were excluded, because in our current treatment strategy these are scheduled as emergency surgeries and inclusion in the study would interfere with this.

Included in the study were patients of 18 years and older who had given their written informed consent. In addition, patients had to be able to pinpoint their drop in VA to a specific 24-hour period in case of a 24-hour to 1 week macular detachment, and to a period of less than one week in case of a macular detachment of one to six weeks. Surgery was performed within 24–72 hours after presentation at the department. The cut-off point of ≤1 week or >1 week was chosen, based on results of previous studies. [11], [14] Patients with macular detachment of more than 6 weeks duration were excluded, because they are considered rare and yield a worse prognosis. [9] Excluded were patients with a bilateral RRD, a history of congenital or acquired pathology with an effect on visual function in one or both eyes, or pathology observed at presentation after their macula-off RRD (i.e. pathology of the cornea, lens, vitreous body, retina (including macula and optic nerve), and scleritis). Patients with a congenital defect in color vision were included. In addition, patients who had a re-detachment during the follow-up period were excluded.

Macula-off RRDs of less than one week duration were scored per day, and of more than one week duration they were scored as 11 days (1–2 weeks duration), 18 days (2–3 weeks duration), 25 days (3–4 weeks duration), 32 days (4–5 weeks duration), and 39 days (5–6 weeks duration), respectively.

We acquired the following patients' characteristics: age, gender, affected eye, ophthalmic history, general history. Using standardised protocols, the refractive error and BCVA using the Early Treatment of Diabetic Retinopathy Study (ETDRS) chart were determined for the affected and fellow (control) eye. [27] All BCVA measurements were converted to logMAR equivalents of ETDRS visual acuity for analysis. Light perception or hand movements were coded as logMAR VA of 3.0.

The height of the macular detachment was determined by optical coherence tomography (OCT) and by ultrasonography using the B-mode scanner at a frequency of 20 MHz.

For OCT measurements, the STRATUS 3000 OCT (Carl Zeiss Ophthalmic System, Dublin, CA) was used. The height of macular detachment was defined as the distance between the outer surface of the photoreceptor layer and the inner surface of the retinal pigment epithelial layer (RPE, second high reflective line). This distance was measured using the analysis program by placing calipers at both surfaces at the foveal dip.

**Figure 1:**
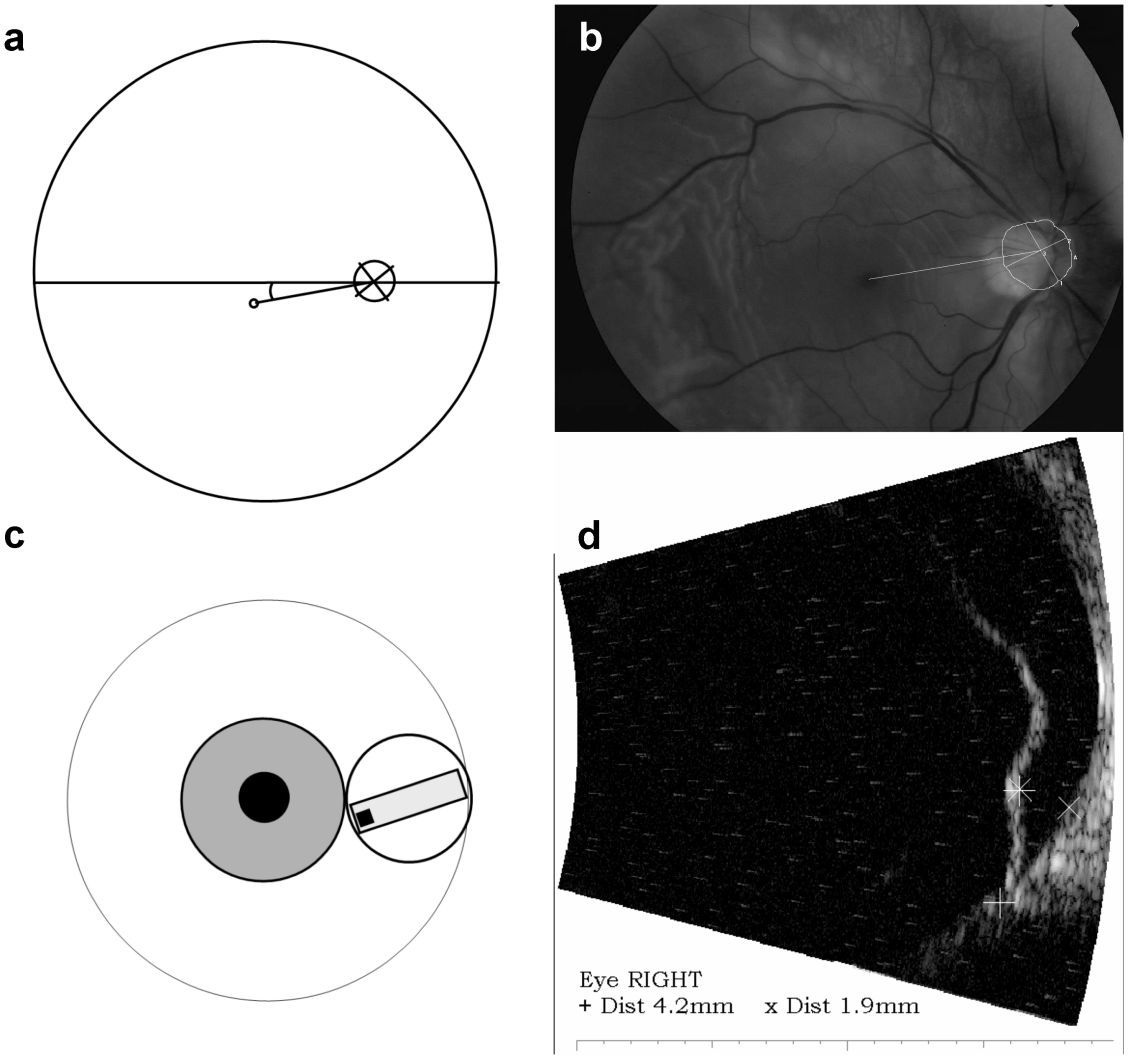
Method to measure the height of the foveal detachment by ultrasonography. **a**. Schematic drawing of the measurement of the distance between the center of the optic nerve head and fovea, and assessment of the angle between their connecting line with the horizon. **b**. Drawing of the measurement of the distance between the center of the optic nerve head (determined by drawing a circle along the outer edge of the optic disc, excluding the area of peripapillary atrophy) and fovea, and assessment of the angle between their connecting line with the horizon made on a fundus photograph. **c**. Schematic position of the ultrasound probe at the correct angle between the horizon and the connecting line between the center of the optic nerve head and the fovea based on the measurement made on the fundus photograph **d**. Measurement of the height of the retinal detachment by ultrasonography at the measured distance between the center of the optic nerve head and the fovea, as obtained by measuring this distance on the fundus photograph.

To measure the height of the detachment at the position of the central macula by ultrasonography, the relative positions of the central macula and the optic nerve head were determined before performing a ultrasonography. For this purpose, digital fundus photographs of both eyes were made using the TRC-50 IX fundus camera (Topcon 9B ltd. UK). On both fundus photographs, the distance between the optic nerve head and fovea was measured using the software package IMAGEnet2000 2.53. The measured distance in the affected eye was used to determine the central position of the macula and at this position the height of the macular detachment was measured by ultrasonography (20 MHz HF long focus B-scan transducer, Quantel Medical Cinescan S, V:5.06)(Figure 1). In those cases (i.e. bullous retinal detachment), in which the measurement of the distance between the macula and optic nerve head could not be performed on the photograph of the affected eye, the measurement of this distance in the fellow eye was used. [32] In each patient, two measurements were made with the patient in an upright position (as this represents the position most patients would have taken for most of the time before presentation during the day) and the average of both measurements was used for further analysis. In addition, in each patient, two measurements were made in a lying position and averaged for analysis.

Most patients (n = 26) had a maximum height of macular detachment in a sitting position. However some had a maximum height when measured in a lying position (n = 19). We hypothesized that the maximum height (independent of position) might be valuable in predicting functional outcome. Therefore, calculations were done again with the mean of the two measurements obtained at the position of maximum height of the macular detachment.

At twelve months postoperatively, we measured BCVA using the ETDRS chart, [27] contrast acuity using the Pelli Robson chart, [26] Farnsworth D-15 saturated, and Lanthoni desaturated color confusion indexes (CCI). [28] All measurements were done in the affected and non-operated fellow (control) eye.

Because of an increased risk of cataract development after trans pars plana vitrectomy, which may influence postoperative measurements, we scored the level of cataract using the lens opacities classification system III (LOCS III) in both eyes at fixed post-operative intervals. [33] In addition, BCVA was assessed at these time points, and in case of a visually significant cataract a cataract extraction was performed before the 12 month measurement.

Depending on the distribution of the variable, either a one-tailed paired Student's t-test (normal distribution) or a Mann-Whitney U test (non-normal distribution) was used to explore statistical differences in visual function. Pearson's (normal distribution) or Spearman's coefficient (non-normal distribution) was used to determine the correlation between measurements of the operated and the fellow eyes. For these analyses, p-values below 0.05 were considered statistically significant. Multivariate forward stepwise regression analyses were performed on the postoperative visual function variables with duration of the macular detachment, height of the macular detachment, age, gender, pre-operative BCVA, and preoperative refractive error as independent variables. For LogMAR BCVA and ln(desaturated CCI), linear regression analysis was used. For log contrast acuity and saturated CCI, ordinal regression analysis was used, since these were not normally distributed.

We also analyzed subgroups of patients with a macular detachment of between ≥24 hours and ≤7 days duration, and of patients with a macular detachment of between >7 days and ≤6 weeks. In these analyses, only the covariates that were significant for the entire group were included. To test whether the effects of the covariates were significantly different between short and long duration of macular detachment, the models were also applied to the entire group with the interaction terms of the significant covariates with an index variable for short or long duration included. A statistically significant p-value for the interaction thus indicates that the regression coefficients differ significantly between the two groups and hence the effect size of the covariate in one group is larger compared to the effect size in the other group.

Because four outcome variables of visual function recovery were tested, we applied a Bonferroni correction for multiple testing and therefore p-values below 0.0125 were considered statistically significant. Data were analysed using SPSS software package, version 16.0 (Chicago, Illinois, USA).

## Results

A total of 56 patients gave their written informed consent. Ten patients were excluded because of re-detachment. One patient deceased during the study period and was therefore excluded from the analysis. In two patients, data on color vision were set to missing because of congenital color blindness.

Table 1 shows the characteristics of our population. The right eye was involved in 22 cases (48.9%) and the left eye in 23 cases (51.1%). A TPPV (n = 39) sometimes combined with an encircling band (n = 22) was most frequently chosen as the primary surgical procedure (86.7%), and in 6 cases a scleral buckling procedure was chosen (13.3%). During the twelve month follow-up period, cataract surgery was performed in 21 out of 29 phakic eyes.

**Table 1:** characteristics of the RRD patients.

	Number (percentage)	Mean age (SD)	scleral buckling/TPPV
Total	45 (100)	61.0(10.6)	6/39
M	34 (75.6)	61.6 (10.5)	2/32
F	11 (24.4)	59.0 (11.0)	4/7
Phakic	29 (64.4)	58.7 (8.9)	6/23
M	21(72.4)	59.2 (8.6)	2/19
F	8 (27.6)	57.5 (9.5)	4/4
Pseudophakic	16 (35.6)	65 (12.3)	0/16
M	13 (81.3)	65.5 (11.9)	0/13
F	3 (18.8)	63 (13.5)	0/3

SD: standard deviation; TPPV: trans pars plana vitrectomy; M: Male F: Female

Due to the fact that macular height often exceeded the maximum value measurable by OCT, OCT measurements could be carried out in only 17/45 patients, whereas ultrasonography measurements could be carried out in all. Therefore, we chose to use the latter technique in our analyses of macular height.

The median duration of the macular detachment was 7 days (range 2–32). The mean height of the macular detachment in sitting position was 1.37 mm. ±1.22 (in two patients only one measurement was taken). We observed no statistically significant difference between mean height in patients treated by buckle surgery (1.38 mm. ±0.74) versus TPPV (1.37 mm. ±1.3) (p = 0.988).

Table 2 provides descriptive data and statistics on pre- and post-operative logMAR BCVA, log contrast acuity, and CCI (saturaté and desaturaté) for operated compared to fellow eyes. It shows that at twelve months postoperatively, all visual function tests are still significantly worse in operated compared to fellow eyes. There was no statistically significant difference between postoperative logMAR BCVA, log contrast acuity and CCI (saturaté and desaturaté), between eyes operated on by buckle or TPPV, nor between phakic and pseudophakic eyes (data not shown). Also, in phakic eyes, no relationship was found between LOCS score and outcomes of any of the above visual function tests (data not shown).

**Table 2:** Tests of visual functioning in operated and non-operated fellow eyes(n = 45).

Visual function	Operated Eyes		Non-operated Eyes		P-value	correlation coefficient
	**Mean**	**SD**	**Mean**	**SD**	**P** *	**R^†^**
Visual acuity 12 month postoperatively (logMAR)	0.31	0.32	0.054	0.12	<0.0005	0.082
	**Median**	**Range**	**Median**	**Range**	**P** **	**R** ‡‡
Pre-operative visual acuity (logMAR)	3.00	0.06–3.00	0.06	−0.20–0.86	<0.0005	0.24
Contrast acuity 12 month postoperatively (log)	1.50	0.15–1.70	1.55	1.25–1.90	0.002	0.19
Color vision CCI saturé 12 month postoperatively***	1.13	1.00–2.66	1.00	1.00–2.06	0.03	0.48
Color vision CCI desaturé 12 month postoperatively***	1.76	1.00–3.20	1.63	1.00–2.59	0.02	0.70

At twelve months postoperatively, all visual function test outcomes are still significantly worse in operated compared to fellow eyes.

*Paired Student's t-test; ^†^ Pearson's correlation coefficient;

**Wilcoxon rank test;

‡‡ Spearman's correlation coefficient,

*** = n = 43, because two colorblind patients were excluded.

Table 3 provides the nominally statistically significant results of the regression analyses on post-operative visual functioning. In the total group, both the duration and the height of the macular detachment were significantly associated with the recovery of visual function. A longer duration of the macular detachment was significantly associated with a worse post-operative CCI saturé (p = 0.0026), and higher LogMAR BCVA (lower Snellen visual acuity) (p = 0.012). A larger height of the macular detachment was significantly associated with a higher post-operative LogMAR BCVA (p = 0.0034). Further, both an older age (Log contrast acuity (p = 1.7×10^−4^)), and a higher pre-operative LogMAR BCVA (log contrast acuity (p = 0.0034) were significantly associated with a worse post-operative contrast acuity.

**Table 3:** Factors influencing recovery of visual function 12 months after surgery.

VISUAL FUNCTION	all patients		≤7 days		>7 days		Interaction*	
	*b*	*p-value*	*b*	*p-value*	*b*	*p-value*	*b*	*p-value*
**LogMAR BCVA** †								
Duration of macular detachment	0.016	**0.012**	0.046	0.082	0.005	0.65	−0.030	0.11
Height of macular detachment	0.10	**0.0034**	0.081	**0.0076**	0.29	*0.016*	0.22	*0.018*
**Log contrast acuity** **								
Age	−0.067	**1.7×10^−4^**	−0.071	**0.0076**	−0.083	**0.0027**	0.0093	0.44
Pre-operative logMAR BCVA	−0.49	**0.0034**	−0.47	*0.036*	−0.62	*0.021*	−0.29	0.35
**CCI saturé^††^**								
Duration of macular detachment	0.081	**0.0026**	0.22	0.29	0.11	**0.0070**	−0.098	0.065
**CCI desaturé** ***								
No significant covariates								

Presented are the effect sizes (a larger b-value represents a greater effect size) and significances (p-values) of the covariates that are statistically significantly associated with visual function measurements for the total group of 45 patients. Subgroup analyses in patients with macula-off RRD of ≤7 days duration (n = 25) and those with macula-off RRD of >7 days duration (n = 20) show that most of the findings observed in the entire patient group remain statistically significant. Significant results after multiple testing correction are shown in bold, nominally significant results (p-value <0.05) are shown in italic.

*Effect size of interaction of covariate with short or long duration of macula-off RRD. A statistically significant p-value for the interaction indicates that the regression coefficients differ significantly between the two groups and hence the effect size of the covariate in one group is larger compared to the effect size in the other group.

† LogMar analyzed with linear regression.

**Log contrast acuity analyzed with ordinal regression with a complementary log-log link function.

‡ CCI saturé analyzed with ordinal regression with a negative log-log link function.

***ln(CCI desaturé) analyzed with linear regression.

b: regression coefficient.

Subgroup analyses in patients with macula-off RRD of ≤7 days duration (n = 25) and those with macula-off RRD of >7 days (n = 20) show that most findings remain (nominally) statistically significant in one or both subgroups. Only height of the macular detachment had a nominally statistically significantly smaller effect (i.e. smaller regression coefficient b) on LogMAR BCVA in patients with a macular detachment of 7 days or less compared to those with a macular detachment of longer than 7 days (p interaction  = 0.018).

Also, duration of macular detachment seemed to have a different effect in the two subgroups,but this difference did not reach statistical significance. Nevertheless, the larger effect size (regression coefficient b) and the lower p-value in the shorter duration group might argue for a more pronounced effect of the duration of macular detachment on recovery of BCVA within the first week of macular detachment compared to after this time point.

For contrast acuity, the effect sizes in the two subgroups did not meet the multiple testing corrected significance threshold, but both were nominally statistically significant. As additionally the interaction was not statistically significant, this suggests that the influence of pre-operative logMAR BCVA on contrast acuity is not affected by the duration of macular detachment.

Finally, calculations with the maximum of the height in sitting or lying position instead of height in sitting position did not yield significant correlations with any of the functional recovery tests (data not shown).

## Discussion

We found that visual recovery after macula-off RRD is affected by both the duration and the height of the macular detachment when measured in sitting position. These factors influence various aspects of visual recovery in different ways. Recovery of visual acuity is decreased in case of a larger height or a longer duration of macular detachment. Recovery of contrast acuity is affected by the age of the patient and the pre-operative LogMAR BCVA. Post-operative saturated color vision is only related to the duration of the macular detachment.

We found that a longer duration of the macular detachment is associated with a worse recovery of visual acuity. This is in line with previous studies, which showed a worse postoperative recovery of visual acuity after longer-standing macula-off RRDs. [2], [9], [11], [14], [18]–[22] In our study, mean post-operative recovery of VA was higher [2], [9]–[13], [14], [22], [34] and the relationship between the duration of the macular detachment and the recovery of VA was a little weaker compared to the results of some others, [9], [13] and somewhat stronger when compared to the study of Ross et al. [34]

Differences between the outcomes of these studies can be explained by differences in study design and studied populations. Some studies were carried out retrospectively, [11]–[12], [34] whilst others, like our own, were carried out prospectively. [10], [13]–[14], [23] Studies may have used different definitions and examination methods. [10]–[14], [23], [34] In- and exclusion criteria were comparable among all studies. [10]–[14], [23], [34] Some studies were larger [10]–[14], [34] than our study and some others. [13], [23] A smaller study population may have led to low power to detect some statistical differences. [13], [23] The follow-up period varied between studies and it is known that postoperative VA can improve in the first postoperative year and sometimes even after one year follow-up. [10]–[14], [23], [31], [34] A follow-up period of one year allowed us to perform a cataract extraction prior to the 12 month measurements, whereas in studies with shorter follow-up periods a visually significant cataract may have caused a lower VA. [10]–[14], [23], [31], [34] In contrast to others, we measured VA as accurately as possible by using ETDRS charts. [10]–[14], [23], [27], [35] In some studies a scleral buckling procedure was performed in all cases, [2], [9], [11], [14], [18]–[22] whereas in our and another recent study, both TPPV and conventional surgery were performed. [10] Finally, older age has been pointed out as associated with a worse recovery. [2], [22] This could have influenced outcomes, since mean [10], [12], [14] or median [34] age differed between studies.

Over the past eighty years, perceptions on the critical period of macular detachment after which visual prognosis may be compromised, have changed from about 1 month in the earliest studies [2], [19] to about 1 week in the more recent ones. [9], [11], [14], [20]–[21] Ross et al. observed no statistically significant difference in post-operative recovery of VA in macula-off RRD patients operated on between 1 to 2 days, 3 to 4 days, and 5 to 7 days after macular detachment. [14] Other recent studies are in line with this and seem to indicate that a surgical delay within this time period may not adversely affect postoperative visual function. [13]–[14], [23] Although we observed a statistically significant relationship between duration of macular detachment and postoperative BCVA in the total study group, significance was lost in our subgroup analyses, in which we looked separately at patients with a macular detachment of ≤7 days and those with a detachment >7 days. However, we did observe that the effect size (regression coefficient b) for the duration of macular detachment was higher and the p-value was lower within the first week of macular detachment compared to after this time point. This probably indicates that our study was underpowered to determine the true effect of delaying surgery within the first week of macular detachment.

In line with others, we identified that a higher macular detachment - when measured in an upright position - is associated with a worse recovery of BCVA. [2], [15], [22], [24], [25] The earliest studies defined macular height in a dichotomous manner as either bullous or shallow by a clinical estimation of submacular fluid. [2], [22] Even by this crude method, a relationship between bullous macular detachment and a worse recovery of VA was observed. [2], [22] Studies using OCT can only measure shallow macular detachments accurately [25] and height might be considered dichotomous in case the height of the detachment surpasses the maximum value measurable by the OCT. [25]

When comparing our results to results of other studies using ultrasonography as a measuring method of macular height, our study identified a stronger relation between macular height and postoperative recovery of BCVA compared to Mowatt et al. [24] This difference may be explained by differences in other variables between both studies, i.e. our study had a larger number of patients, a shorter median duration of the macular detachment, a worse pre-operative VA, a lower mean height of the macular detachment, an older age, a longer follow-up period, and a more frequent use of TPPV as the surgical technique. [24] In addition, we identified the strongest relation (lower p-value) between macular height and postoperative recovery of BCVA in patients with a macula-off RRD of shorter duration and the largest effect size (regression coefficient b) between these factors in the longer duration group. Also in contrast to Mowatt et al. [24] - we observed that a higher macular detachment at presentation is related to worse postoperative recovery of VA in macula-off RRD of shorter duration, which is in line with observations made by Ross et al. in a larger study. [14]

We found no relationship between both the duration and the height of the macular detachment and postoperative recovery of contrast acuity, a relationship previously observed by others. [13] Previous studies found that contrast acuity scores after macula-off RRD are lower when compared to a control group of similar age [35] and to fellow eyes, [13] which is in line with our findings. In our study, an older age at presentation was highly correlated with a worse postoperative contrast acuity, a relationship that was previously described for VA. [2], [22]

Although previously pointed out as an important factor for postoperative recovery in macula-off RRD, [2], [18] we did not identify pre-operative VA as a significant predictor of postoperative recovery of LogMAR BCVA. We did identify a relationship between pre-operative VA and postoperative contrast acuity, which – as far as we are aware – was not previously reported.

We found that a longer duration of the macular detachment is associated with a worse recovery of color vision. This is in line with previous studies on postoperative color vision after macula-off RRD.[13,37] In addition, the longer the duration of the macular detachment, the deeper the color defect was, [13] particularly if the duration of the macular detachment exceeded 7 days. Kreissig et al. found postoperative color vision disturbances in half of their patients, in particular, in older patients. [36] Özgür and Esgin found significantly more color vision defects in macula-off RRD eyes compared to their healthy fellow-eyes, in a comparable study group. [13] We observed the strongest relation with a worse recovery of color vision in macula-off RRD of longer duration.

*O*ur study highlights important factors related to the postoperative recovery of visual function, however there are several limitations. First, there could have been a selection bias, because highly motivated patients may have participated in this study. Second, the determination of the onset of the macular detachment remains subjective, and may not always be correct, especially in shallow detachments where a decrease in VA may be small and pass unnoticed for a prolonged period of time. Further, we measured macular height in an upright position assuming that this would best represent the position of the macula in daytime conditions. However, the height of a retinal detachment may vary depending on posture and this variation has not been taken into account. The absence of a significant correlation between the maximum height of detachment in sitting or lying position and functional recovery makes the value of this factor at least questionable. Finally, the studied population is relatively small and thus our study may have been underpowered to detect some possible relations.

In conclusion, we found that visual recovery after macula-off RRD is affected by both the duration and the height of the macular detachment. The seemingly strong relationship between the height of macular detachment and recovery of BCVA might indicate that posturing of macula-off RRD patients could be valuable. However, at present, we have to be critical with regard to this factor, which may have been highly variable over time. Therefore, we would recommend to evaluate the effect of posturing on the height of macular detachment and on visual recovery in a future study.

## References

[1] van de Put MAJ, Hooymans JM, Los LI (2013) The Dutch Rhegmatogenous Retinal Detachment Study Group (2013) The incidence of rhegmatogenous retinal detachment in the Netherlands. Ophthalmology 120: 616–622. 23218185 10.1016/j.ophtha.2012.09.001

[2] Tani PT, Robertson DM, Langworthy A (1981) Prognosis for central vision and anatomic reattachment in rhegmatogenous retinal detachment with macula detached. Am J Ophthalmol 92: 611–620. 7304687 10.1016/s0002-9394(14)74651-3

[3] Pastor JC, Fernández I, Rodríguez de la Rúa E, Coco R, Sanabria-Ruiz Colmenares MR, et al (2008) Surgical outcomes for primary rhegmatogenous retinal detachments in phakic and pseudophakic patients: the Retina 1 Project – report 2. Br J Ophthalmol 92: 378–382. 18303159 10.1136/bjo.2007.129437

[4] D'Amico DJ (2008) Primary retinal detachment. N Engl J Med 359: 2346–2354. 19038880 10.1056/NEJMcp0804591

[5] Chignell AH, Fison LG, Davies EW, Hartley RE, Gundry MF (1973) Failure in retinal detachment surgery. Br J Ophthalmol 57: 525–530. 4743910 10.1136/bjo.57.8.525 PMC1215082

[6] Rachal WF, Burton TC (1979) Changing concepts of failure after retinal detachment surgery. Arch Ophthalmol 18: 415–429. 10.1001/archopht.1979.01020010230008 420635

[7] Sharma T, Challa JK, Ravishankar KV, Murugesan R (1994) Scleral buckling for retinal detachment: predictors for anatomic failure. Retina 14: 338–343. 7817027 10.1097/00006982-199414040-00008

[8] Grizzard WS, Hilton GF, Hammer ME, Taren D (1994) A multivariate analysis of anatomic success of retinal detachments treated with scleral buckling. Graefes Arch Clin Exp Ophthalmol 232: 1–7. 8119596 10.1007/BF00176431

[9] Burton TC (1982) Recovery of visual acuity after retinal detachment involving the macula. Trans Am Ophthalmol Soc 80: 475–497. 6763802 PMC1312277

[10] Mitry D, Awan MA, Borooah S, Syrogiannis A, Lim-Fat C, et al.. (2012) Long term visual acuity and the duration of macular detachment: findings from a prospective population based study. Br J Ophthalmol 1–4. 10.1136/bjophthalmol-2012-302330 23159447

[11] Diederen RMH, La Heij AC, Kessels AGH, Goezinne F, Liem AT, et al (2007) Scleral buckling surgery after macula-off retinal detachment; worse visual outcome after more than 6 days. Ophthalmology 114: 705–709. 17194479 10.1016/j.ophtha.2006.09.004

[12] Hassan TS, Sarrafizadeh R, Ruby AJ, Garretson BR, Kuczynski B, et al (2002) The effect of duration of macular detachment on results after the scleral buckle repair of primary, macula-off retinal detachments. Ophthalmology 109: 146–152. 11772595 10.1016/s0161-6420(01)00886-7

[13] Özgür S, Esgin H (2007) Macular function of successfully repaired macula-off retinal detachments. Retina 27: 359–364. 10.1097/01.iae.0000243063.22337.ae 17460592

[14] Ross WH, Kozy DW (1998) Visual recovery in macula-off rhegmatogenous retinal detachments. Ophthalmology 105: ; 2149–2153. 10.1016/S0161-6420(98)91142-3 9818620

[15] Machemer R (1968) Experimental retinal detachment in the owl monkey. II. Histology of the retina and pigment epithelium. Am J Ophthalmol 66: 396–410. 4970986 10.1016/0002-9394(68)91523-7

[16] Mervin K, Valter K, Maslim J, Lewis G, Fisher S, et al (1999) Limiting photoreceptor death and deconstruction during experimental retinal detachment; the value of oxygen supplementation. Am J Ophthalmol 128: 155–164. 10458170 10.1016/s0002-9394(99)00104-x

[17] Lewis GP, Talaga KC, Linberg KA, Avery RL, Fisher SK (2004) The efficacy of delayed oxygen therapy in the treatment of experimental retinal detachment. Am J Ophthalmol 137: 1085–1095. 15183794 10.1016/j.ajo.2004.01.045

[18] Gundry MF, Davies EWG (1974) Recovery of visual acuity after retinal detachment surgery. Am J Ophthalmol 77: 310–314. 4813306 10.1016/0002-9394(74)90735-1

[19] Reese AB (1937) Defective central vision following successful operations for detachment of the retina. Am J Ophthalmol 20: 591–598.

[20] Jay B (1965) The functional cure of retinal detachments. Trans Ophthalmol Soc UK 85: 101–110. 5227173

[21] Davies EWG (1972) Factors affecting recovery of visual acuity following detachment of the retina. Trans Ophthalmol Soc UK 92: 335–342. 4515518

[22] Kreissig I (1977) Prognosis of return of macular function after retinal reattachment. Mod Probl Ophthalmol 18: 415–429. 876086

[23] Ross WH, Lavina A, Russel M, Maberley D (2005) The correlation between height of macular detachment and visual outcome in macula-off retinal detachments of ≤ 7days' duration. Ophthalmology 112: ; 1213–1217. 10.1016/j.ophtha.2005.01.040 15921745

[24] Mowatt L, Tarin S, Nair RG, Menon J, Price NJ (2010) Correlation of visual recovery with macular height in macula-off retinal detachments. Eye 24: 323–327. 19390562 10.1038/eye.2009.74

[25] Lecleire-Collet A, Muraine M, Menard JF, Brasseur G (2005) Predictive visual outcome after macula-off retinal detachment surgery using optical coherence tomography. Retina 259: 44–53. 10.1097/00006982-200501000-00006 15655440

[26] Mantyjarvi M, Laitinen T (2001) Normal values for the Pelli-Robson contrast sensitivity test. J Cataract Refract Surg 27: 261–266. 11226793 10.1016/s0886-3350(00)00562-9

[27] Rosser DA, Cousens SN, Murdoch IE, Fitzke FW, Laidlaw DA (2003) How sensitive to clinical change are ETDRS logMAR visual acuity measurements? Invest Ophthalmol Vis Sci 44: 3278–3281. 12882770 10.1167/iovs.02-1100

[28] Bowman KJ (1982) A method for quantitative scoring of the Farnsworth panel D-15. Acta Ophthalmol 60: 907. 6984998 10.1111/j.1755-3768.1982.tb00621.x

[29] Wilkinson CP (2009) Mysteries regarding the surgically reattached retina. Trans Am Ophthalmol 107: 55–59. PMC2814586 20126482

[30] Minihan M, Tanner V, Williamson TH (2001) Primary rhegmatogenous retinal detachment: 20 years of change. Br J Ophthalmol 85: 546–548. 11316713 10.1136/bjo.85.5.546 PMC1723977

[31] Liem AT, Keunen JE, van Meel GJ, van Norren D (1994) Serial foveal densitometry and visual function after retinal detachment surgery with macular involvement. Ophthalmology 101: 1945–1952. 7997333 10.1016/s0161-6420(94)31078-5

[32] Van de Put MAJ, Nayebi F, Croonen D, Nolte IM, Japing WJ, et al (2013) Design and validation of a method to determine the position of the fovea by using the nerve-head to fovea distance of the fellow eye. PlosOne 8: E62518. 10.1371/journal.pone.0062518 PMC3646827 23667483

[33] Chylack LT Jr, Wolfe JK, Singer DM, Leske MC, Bullimore MA, et al (1993) The Lens Opacities Classification System III. The Longitudinal Study of Cataract Study Group. Arch Ophthalmol 111: 831–836. 8512486 10.1001/archopht.1993.01090060119035

[34] Doyle E, Herbert EN, Bunce C, Williamson TH, Laidlaw DAH (2007) How effective is macula-off retinal detachment surgery. Might good outcome be predicted. Eye 21: 534–540. 16456590 10.1038/sj.eye.6702260

[35] Anderson C, Sjöstrand J (1981) Contrast sensitivity and central vision in reattached macula. Acta Ophthalmol 59: 161–169. 7257734 10.1111/j.1755-3768.1981.tb02975.x

[36] Kreissig I, Lincoff B, Witassek B, Kolling G (1981) Color vision and other parameters of macular function after reattachment. Dev Ophthalmol 2: 77–85. 7262430 10.1159/000395308

